# Phylogeny and adaptive evolution of the brain-development gene microcephalin (*MCPH1*) in cetaceans

**DOI:** 10.1186/1471-2148-11-98

**Published:** 2011-04-14

**Authors:** Michael R McGowen, Stephen H Montgomery, Clay Clark, John Gatesy

**Affiliations:** 1 Department of Biology, University of California, Riverside, Riverside, CA 92521 USA; 2 Center for Molecular Medicine and Genetics, Wayne State University School of Medicine, 540 E. Canfield Ave., Detroit, MI 48201 USA; 3 Department of Zoology, University of Cambridge, Downing Street, Cambridge, CB2 3EJ, UK

## Abstract

**Background:**

Representatives of Cetacea have the greatest absolute brain size among animals, and the largest relative brain size aside from humans. Despite this, genes implicated in the evolution of large brain size in primates have yet to be surveyed in cetaceans.

**Results:**

We sequenced ~1240 basepairs of the brain development gene microcephalin (*MCPH1*) in 38 cetacean species. Alignments of these data and a published complete sequence from *Tursiops truncatus *with primate *MCPH1 *were utilized in phylogenetic analyses and to estimate ω (rate of nonsynonymous substitution/rate of synonymous substitution) using site and branch models of molecular evolution. We also tested the hypothesis that selection on *MCPH1 *was correlated with brain size in cetaceans using a continuous regression analysis that accounted for phylogenetic history. Our analyses revealed widespread signals of adaptive evolution in the *MCPH1 *of Cetacea and in other subclades of Mammalia, however, there was not a significant positive association between ω and brain size within Cetacea.

**Conclusion:**

In conjunction with a recent study of Primates, we find no evidence to support an association between *MCPH1 *evolution and the evolution of brain size in highly encephalized mammalian species. Our finding of significant positive selection in *MCPH1 *may be linked to other functions of the gene.

## Background

The human brain is arguably one of the most remarkable adaptations in the history of life. Compared to other mammals, the human lineage has undergone a massive expansion in relative brain and forebrain size, cortical surface area, and overall cognitive ability [1]. However, many other vertebrates exhibit increased relative brain and forebrain sizes, as well as complex social and cognitive behaviours. For example, odontocete cetaceans (toothed whales) have some of the largest brains relative to their body mass among extant mammals [2]. Relative brain size in some odontocete species is greater than non-human primates [3], and in absolute terms, the giant sperm whale (*Physeter macrocephalus*) has the largest brain of any living organism at a maximum of 10 kg [4]. According to some researchers, high relative brain or forebrain sizes are positively correlated with indices of cognition or "intelligence" [1,5], although this association has been criticized in the literature [6,7].

**Figure 1:**
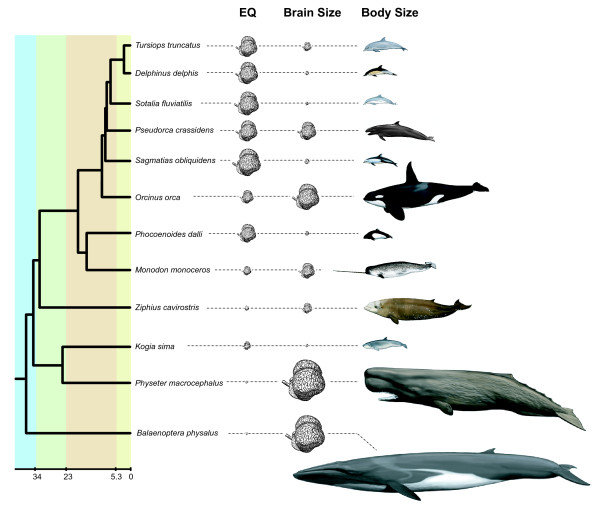
**Variation in absolute brain size, relative brain size (EQ = encephalization quotient), and body size in cetaceans in a phylogenetic context**. Representatives from seven cetacean families are shown and are scaled to body length. EQ and absolute brain size are indicated to the right of each species name by drawings of brains. The width of each brain is proportioned to the value of EQ or brain weight for each species. For scale, EQ of *Physeter macrocephalus *is 0.58 and of *Tursiops truncatus *is 4.02. Brain weight of *Physeter macrocephalus *is 8.00 kg and of *Tursiops truncatus *is 1.76 kg. Phylogenetic relationships and approximate divergence times are from [42]. For the timescale, dark green = Eocene, light green = Oligocene, orange = Miocene, and yellow = Plio-Pleistocene. EQ and brain weights for all species in our analysis are shown in Supplemental Table S2.

Among extant cetacean species, absolute and relative brain size vary widely (Figure 1). There is some evidence that a large shift towards increased brain size took place near the base of Odontoceti (toothed whales), and a further increase in Delphinoidea, the group that includes Delphinidae (oceanic dolphins) among others [8]. Delphinids display the greatest encephalization and the most complex behavior among cetaceans [9,10]. The evolution of large brains in odontocetes has been linked to their intricate behavioral repertoire and to their use of echolocation [11], which requires production and processing of high frequency sounds to perceive spatial relationships in the surrounding liquid environment [2]. Odontocete cetaceans also are distinguished by indices of complex cognition that are convergent with many primate species [9,12]. Some researchers have proposed that odontocetes evolved large brains for thermoregulation and are not as socially and behaviorally advanced as primates [13], however this has been contested [10].

In primates, researchers have documented the association of six genes (*MCPH1, ASPM, CDK5RAP2, CENPJ, STIL, WDR62*) with the human congenital disorder of primary microcephaly, a disease marked by a two-thirds reduction in brain size and moderate to severe mental retardation [14-22]. All six microcephaly genes identified thus far are involved in control of the neural cell cycle and/or centrosome function, and therefore have a potentially large impact on the proliferation of neural precursor cells [23,24]. Comparative analyses of protein-coding DNA sequences from primates suggest that these genes have evolved adaptively in primates [14-19,25].

Multiple recent studies have revealed the importance of one of these genes, microcephalin (*MCPH1*), in maintaining genomic stability through mediation of the response to double-strand DNA breakage and regulation of chromosome condensation in the cell cycle [26-31]. *MCPH1 *is expressed in multiple tissues including those of the brain, liver, and kidney, but shows particularly high expression in neural progenitor cells of the forebrain [32]. Microcephaly genes may play a role during development in switching between symmetric and asymmetric mitosis of neural progenitor cells to produce neurons in the cerebral cortex [24,33]. Specifically, microcephalin may affect the first stage of neural cell division, with decreased MCPH1 function causing premature mitotic entry, eventually leading to a reduced pool of neural progenitor cells [24]. In addition, the developing neuronal tissue in the brain seems particularly vulnerable to apoptosis as a result of DNA damage caused by double-strand breaks, thus potentially bringing about a decrease in viable neurons for individuals with impaired MCPH1 function [34,35].

Although some lineages of whales and dolphins have experienced profound evolutionary increases in relative brain size, selection on microcephaly genes has not been investigated in detail within Cetacea. Several researchers have indicated the potential value of comparisons between brain genes of primates and other large-brained species for understanding processes underlying neurological novelty in primates including humans [23,33,36]. Here we sequenced a large segment of microcephalin (*MCPH1*) from 38 cetacean species, including 34 toothed whales and four baleen whales, and compared these data with published sequences from primates, which have convergently evolved large brain size. We also downloaded complete *MCPH1 *protein-coding sequences from Ensembl, including representatives of major primate lineages as well as the common bottlenose dolphin, *Tursiops truncatus*. We enumerated patterns of site- and branch-specific selection in cetacean *MCPH1 *and compared these estimates with patterns of selection intensity in primates and other mammals. We also explicitly tested the hypothesis that brain size is positively correlated with the intensity of selection at the nucleotide level in *MCPH1 *following the approach taken by [25].

## Methods

### Sampling and DNA Amplification

We sampled 38 species of cetaceans from nine families; twenty-three species belonged to Delphinidae, a family that includes the highest relative brain sizes as measured by the encephalization quotient (EQ) [10]. We also included members of Mysticeti (baleen whales) and Physeteridae (giant sperm whale) that represent taxa with the largest absolute brain sizes that have ever evolved [4,9,10]. All cetacean taxa included in this study are listed in Additional File 1: Table S1.

We designed primers for exon 8 of *MCPH1 *from an alignment of sequences from the 2.0 build of the *Bos taurus *(domestic cow) genome and genomic data for *Tursiops truncatus *available via Genbank. Exon 8 consists of the highly variable inter-BRCT domain sequence (IBS) and makes up approximately half of the coding region of *MCPH1*. The IBS region shows evidence of positive selection on the lineage leading to humans [16,19]. A small portion of intron 7 and most of exon 8 (total amplicon = ~1237 basepairs [bp]) were PCR amplified using MCPH1INT7F1 (5' GCT TTA TCA CGT TAT GGG CGG AC 3') or MCPH1INT7F2 (5' GCT TTA TCA CGT TAT GGG CGG ACT G 3') in the forward direction and MCPH1EX8R1 (5' GAG AGA CCA GTA AAG GAG GTT CAC 3'), MCPH1EX8R2 (5' AGG AGG TTC ACA TAC TTT CAC TAC 3'), or MCPH1RSeq2 (5' CGG GAG AAA AGT AAT CAT CG 3') in the reverse direction. PCR products were sequenced using the above primers, as well as MCPH1F1 (5' AAA ACG AGA AGT GTC CGT CCG C 3'), MCPH1F2 (5' CCT GTC TGC TAC GCC ATC TGT AAC 3'), MCPH1FSeq3 (5' TTT CCA GGA GAG AGA GGA CC 3'), MCPH1R1 (5' TTT CCA CAT CCC AGT CGC CTA C 3'), and MCPH1RSeq1 (5' TCT CCT TGA GAT TAT CGG G 3'). PCR was performed using 1 μl template DNA, 100 pmol of each primer, 1X AccuPrime PCR Buffer I (Invitrogen), and 1 unit AccuPrime *Taq *DNA Polymerase High Fidelity (Invitrogen) in a 50 μl reaction. PCR conditions consisted of 45 cycles of 1 min denaturation at 94°C, 1 min annealing at 58°C, and 1 min elongation at 68°C. All new sequences were deposited in Genbank (accession numbers HQ873570-HQ873608).

### Data Set Compilation and Alignment

Three data sets were assembled. To investigate selection pressure over the whole gene, the "whole-gene" data set was compiled using complete *MCPH1 *coding sequences downloaded via Ensembl (*Homo, Pan, Pongo, Macaca, Callithrix, Tupaia, Rattus, Mus, Canis, Equus, Bos*, and the delphinid cetacean, *Tursiops*). Species were selected due to phylogenetic position, completeness, and quality of available sequence. We also compiled a data set consisting of sequences derived from exon 8 and a small segment of intron 7 (the "exon 8" data set), including those sequenced here and sequences downloaded for a wider range of species, largely primates, from the Ensembl and Genbank databases. Genbank accession numbers for all downloaded sequences are listed in Additional File 1. The third data set ("reduced exon 8") consisted of a reduced set of taxa that excluded sequences that were less than 90% complete (e.g., *Balaenoptera acutorostrata, Kogia sima*) and sequences from species belonging to Monodontidae due to the presence of a stop codon near the end of the sequence (see Additional File 1). Sequences for all data sets were aligned using CLUSTAL W [37] with a gap-opening penalty of 10 and a gap-extension penalty of 1. Exonic indels were multiples of 3 bp and were adjusted by eye to reflect the open reading frame.

### Phylogenetic Analyses

Please see Additional File 1 for a full account of phylogenetic methods.

### Tests for Positive Selection and Selective Constraints

Positive selection acting on the complete coding sequence of *MCPH1 *was examined by estimating ω (*dN/dS*, the ratio of the rate of nonsynonymous substitution to the rate of synonymous substitution) using the site models in the codeml program of PAML 4.0 [38]. An unrooted species tree of Boreoeutheria [39-41] was used as an input tree with Rodentia, Primates + Scandentia, and Laurasiatheria positioned as a basal polytomy. Model M1a (nearly-neutral: ω_0 _< 1, ω_1 _= 1) was compared to M2a (positive selection: ω_0 _< 1, ω_1 _= 1, ω_2 _> 1) and M8a (nearly neutral; beta distribution: 0 < ω_0 _< 1 and ω_1 _= 1) was compared to model M8 (positive selection: beta distribution: 0 < ω_0 _< 1 and ω_1 _> 1) by performing likelihood ratio tests (LRTs) and assessing their significance using a χ^2 ^distribution (two degrees of freedom [df] for M1a vs. M2a; one df for M8 vs. M8a). For the comparison of M8 vs. M8a, we halved the p-value as suggested by [38]. A Bayes Empirical Bayes (BEB) analysis was implemented to calculate posterior probabilities of positively selected sites using the M2a and M8 models as described in [38]. An individual site was considered to have undergone positive selection (ω > 1), if the posterior probability was ≥0.95. Variation in ω among branches was examined using the free-ratio model in which each branch of the tree was given a separate ω-value. The fit of the free-ratio model was compared to model M0 in which all branches in the tree were assigned the same ω value using the LRT with 20 degrees of freedom for the whole *MCPH1 *data set.

To further investigate selection on exon 8 of *MCPH1*, for which 38 cetacean species were sequenced (Additional File 1: Table S1), all intronic sequence was deleted, as well as sequence downstream of a stop codon found near the 3' end of exon 8 in monodontids (see Additional File 1). All "exon 8" analyses were implemented using a species tree and also a gene tree derived from phylogenetic analysis of the *MCPH1 *data. The species tree employed was a composite gathered from several sources [39-42]; the gene tree for PAML analyses was the optimal topology recovered by maximum likelihood (ML) analysis and was consistent with the 50% majority rule consensus of Bayesian trees. In both the species tree and the gene tree, *Loxodonta*, Euarchontoglires, and Laurasiatheria were treated as a basal trichotomy. The ratio ω was then estimated in the codeml program of PAML 4.0 [38], as described above for the complete coding sequence of *MCPH1*. Separate analyses were conducted using the site models for all mammals, cetaceans only, odontocetes only, delphinids only, mysticetes only, primates only, and all mammals excepting cetaceans and primates. By analyzing various subclades of mammals using the site models, the goal was to determine whether evidence for positive selection is a general feature of *MCPH1 *in mammals, or is instead restricted to only certain mammalian lineages. In addition, analyses using the species tree and gene tree were also conducted for the "reduced exon 8" data set (see above).

For the taxon-rich exon 8 alignment, ω was also estimated for individual branches and groups of branches [38]. Several branch model analyses were conducted including a free-ratio model (all branches separate), two-ratio models in which one branch was given a separate ω (repeated for the branch leading to the last common ancestor [LCA] to each of the following groups: Cetacea, Mysticeti, Odontoceti, Delphinoidea, Delphinidae, as well as each of the terminal branches leading to *Physeter *and *Orcinus*), and two-ratio models in which one stem-based clade was given a separate ω (repeated for Cetacea, Mysticeti, Odontoceti, Delphinoidea, and Delphinidae). All models listed above were tested against a one-ratio model (M0) using LRTs and 1 df. Branch models with *p *≤ 0.004 (0.05/13) after Bonferroni correction for multiple tests were interpreted as having a significantly different ω on the "foreground" branches of interest in comparison to the "background" ω on all remaining branches of the tree. Comparisons between background branches and foreground branch(es) at the base of lineages or within whole clades were conducted to test whether the pattern of selection on these branches was significantly distinct from the rest of the tree. Branches were selected for comparison due to their proposed relation to an evolutionary change in relative and/or absolute brain size. For example, in the case of Odontoceti and Delphinoidea, Marino et al. [8] proposed that these clades mark shifts associated with increases in relative brain size. Delphinidae was selected due to the high relative brain size of multiple species within the clade [8]. The branches leading to Mysticeti, *Physeter, *and *Orcinus *were also tested because these lineages terminate at species with large absolute brain sizes [8,9] (Figure 1, Additional File 1: Table S2). For all PAML branch models, both a species tree and the *MCPH1 *gene tree were used as input trees.

### Variation of *MCPH1 *within Cetacean Species

We recorded heterozygous sites for each cetacean *MCPH1 *sequence that was generated in this study. Sites in sequencing chromatograms that showed nearly equal height peaks for two different bases at the same position were considered true heterozygous sites (i.e., due to divergent alleles that were PCR-amplified from that specimen). The position (first, second, or third codon) and the nature (synonymous or nonsynonymous, transition or transversion, etc.) of change at each heterozygous site were noted, and this variation within species was compared to the pattern of nucleotide substitution in *MCPH1 *between cetacean species. For *Tursiops truncatus *and *Delphinus capensis*, we compared sequences derived from two individuals of each species and checked for intraspecific variation in *MCPH1*.

### Analysis of Associations Between ω and Phenotype

To test prior hypotheses of association between ω and phenotype, we compiled data on absolute brain and body mass for 27 cetaceans for which we had molecular data; these traits vary widely among extant cetaceans (Figure 1). Absolute brain and body mass data came from multiple, previously published sources [8,9,43,44]. For *Platanista minor*, we used measurements derived from the very close relative, *Platanista gangetica *[42,43]. Because some body masses above came from individual animals and may not represent the full size range of a particular species, we also gathered data on maximum body size from species accounts in [45]. In addition, a measure of relative brain mass, the encephalization quotient (EQ), was calculated for each species using a standard allometric equation of mammalian brain mass vs. body mass from [1]: EQ = brain mass/0.12 (body mass)^0.67^. Absolute brain size, body size, maximum body size, and EQ were then log-transformed for statistical analysis (see below). Morphological data were deposited online in Supplemental Table S2.

We used the method of [25] to assess associations between ω and various phenotypic variables: absolute brain mass, absolute body mass, maximum body mass derived from [45], and EQ. For each of the 27 cetacean species that were scored for phenotypic data (see above), we calculated the average "root-to-tip" ω along branches extending from the last common ancestor of Cetacea to each extant cetacean species in our dataset. This approach has the advantage of producing a measure of selection that takes the entire evolutionary history of a lineage from a common ancestor into account and which is a property of the species tips in a way that is more comparable with extant phenotypes. This procedure also negates the issue of temporal effects on ω [25]. Root-to-tip ω values were estimated in PAML 4.0 [38] using a 2-rate branch model and the species tree of [42]. Phylogenetically controlled regression analyses of log-transformed root-to-tip ω versus each log-transformed morphological variable were performed using BayesTraits [46,47] and the time-calibrated tree of [42] to explicitly test for gene-phenotype associations. The significance of the regression analyses was determined using a one-tailed *t*-test in the positive direction, in order to test the hypothesis that there is a positive relationship between estimated selection pressure on *MCPH1 *(ω) and the phenotypic variables as in [25]. Each regression was performed across all cetaceans and just within the Odontoceti.

In addition to the EQ, we also explored two other methods of assessing relative brain size. In the first method, we calculated residuals from a regression between brain and body mass and used these in a subsequent regression analysis with root-to-tip ω. In the second method, we performed a multiple regression of brain and body mass with root-to-tip ω. Both of these approaches produced similar results to the regression using the EQ, and are not shown here.

## Results

### Characterization and Phylogenetic Analysis of *MCPH1*

**Figure 2:**
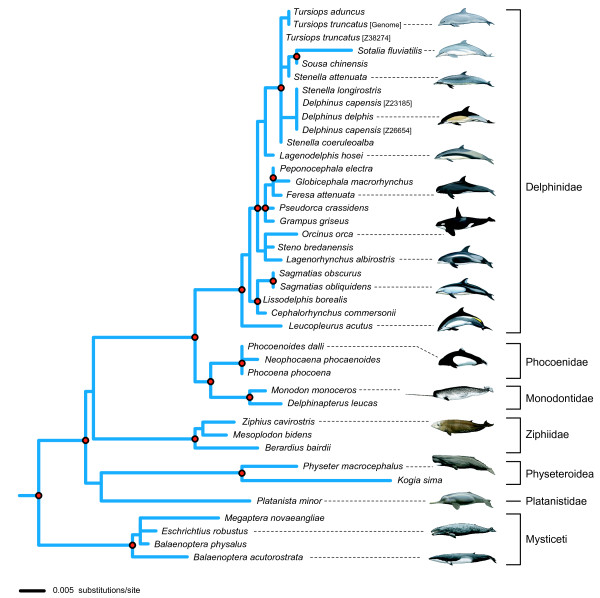
**Relationships among cetaceans in the maximum likelihood (ML) gene tree for *MCPH1 *based on the exon 8 data set**. The phylogram of the whole tree that includes Primates and other terrestrial mammals is presented in Additional file 1, Figure S2. The cetacean part of the tree is illustrated here and shows well-supported nodes within Cetacea (red dots = ML bootstrap ≥70% and Bayesian posterior probability (with and without indels) ≥ 0.95). Higher level taxa are delimited by brackets to the right.

The alignment of the entire protein-coding region of *MCPH1 *consisted of 2571 bp with 31 indels that were in frame and 3-33 bp (multiples of three) in length. Of the complete sequences, protein translations ranged from 822 amino acids (aa) in length in *Mus *to 842 aa in *Callithrix*. Phylogenetic analyses were performed using the exon 8 data set. The topology of the ML tree and the Bayesian consensus trees were congruent (Figure 2; Additional File 1: Figure S1, Figure S2). Most higher-level relationships among mammalian orders and suborders were consistent with those of large comprehensive data sets [39,40]. Many higher-level relationships within Cetacea supported by *MCPH1 *(Figure 2) were congruent with the supermatrix of [42]; however, some relationships differed, especially within Delphinidae. For a more detailed description of the alignment and phylogenetic results, see Additional File 1.

### Molecular Evolution of *MCPH1*

**Figure 3:**
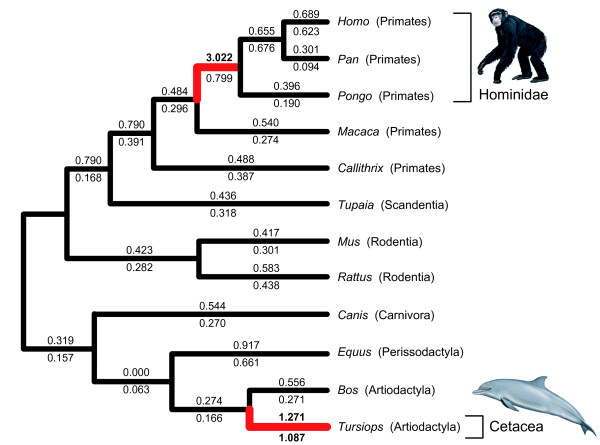
**Selection intensity estimates for species with complete *MCPH1 *sequences**. Phylogenetic relationships are derived from [40,41]. The ω estimates for individual branches according to the "free ratio model" are shown. ω values for the entire protein-coding region of *MCPH1 *are above branches, and ω values that were estimated after exclusion of exon 8 are shown below branches. Red branches mark lineages with ω > 1. The branch that terminates at the cetacean, *Tursiops*, was the only branch with ω > 1 for the whole *MCPH1 *gene and after removal of the highly variable exon 8 from analysis.

For the *MCPH1 *whole gene data set, site models that incorporated positive selection (M2a and M8) were significantly better fits to the data (p < 0.001) than corresponding nearly neutral models (M1a and M8a). Overall, model M2a assigned 3.43% of codons to the class of positively selected sites (ω = 2.334), and model M8 assigned 12.40% to the positive selection class (ω = 1.644). Both models M2a and M8 yielded three sites that had high probabilities (≥0.95 in BEB analysis) of belonging to the class of sites with ω > 1 (308, 398, and 521), all of which were located in the IBS region between the BRCA1 C-terminal (BRCT) domains. None of the three positively selected sites showed evidence of convergence in amino acid sequence between primates and cetaceans. Analyses using branch models revealed that the free ratio model, in which each branch had a unique ω, was significantly better (*p*< 0.0001) than the model in which all branches were constrained to have the same ω. The free-ratio model revealed two branches with ω > 1: the branch leading to the LCA of Hominidae and the terminal branch leading to the bottlenose dolphin *Tursiops *(Figure 3). The greatest ω was on the branch leading to the LCA of Hominidae (ω = 3.022; 12.1 nonsynonymous substitutions and 1.6 synonymous substitutions), and evidence of ω > 1 along this branch agrees with other studies [16,19]. The ω for the terminal branch leading to *Tursiops *(1.271; 175.9 nonsynonymous substitutions and 54.4 synonymous substitutions) is marginally greater than the value expected for a complete absence of selective constraints (ω = 1.000).

Using the coding sequences in the more taxon-rich exon 8 data set of all mammals, both site models incorporating positive selection (M2a and M8) again were significantly better (both p < 0.001) than corresponding models that only incorporate nearly neutral evolution (M1a and M8a; Table 1); This was true whether the species tree (Table 1) or the *MCPH1 *gene tree (Additional File 1: Table S3) was used (in the remainder of the paper, we refer primarily to results using the species tree; gene tree models were highly consistent with results using the species tree). The class of sites with ω > 1 for the M8 model (14.0% of sites) was characterized by an ω that was significantly greater than 1 (ω = 1. 721). M2a and M8 identified one and seven sites, respectively, with an ω > 1 using the BEB method (Table 1). Among sites identified as having an ω > 1, parallel changes in amino acid sequence were not shared between any cetacean and any primate. The mean ω for sites in Model M2a was ω = 0.858 for exon 8. The reduced data set produced similar results (not shown) and identified the same sites under positive selection.

**Table 1:** Results for site model analyses using the species tree derived from multiple sources [39-42]

		Avg.	Prop. sites	ω sites with	Sites under positive selection
Model	-ln *L*	ω	ω > 1	ω > 1	(p > 0.95, BEB)
*All mammals:*					
M1a	15880.84	0.757			
M2a	15869.046	0.858	0.07	2.063	362
M8	15863.642	0.829	0.14	1.721	112, 132, 172, 205, 209, 247, 362
M8a	15877.044	0.737			
M1a v. M2a: df = 2, -2Δln*L *= 23.587, **p < 0.001**					
M8 v. M8a: df = 1, -2Δln*L *= 26.804, **p < 0.001**					
					
*All cetaceans:*					
M1a	3913.4037	0.7975			
M2a	3903.628	0.9548	0.021	6.767	89, 362
M8	3903.6499	0.9458	0.072	6.564	89, 362
M8a	3913.4114	0.8017			
M1a v. M2a: df = 2, -2Δln*L *= 19.551, **p < 0.001**					
M8 v. M8a: df = 1, -2Δln*L *= 19.523, **p < 0.001**					
					
*All odontocetes:*					
M1a	3519.5486	0.7351			
M2a	3514.0255	0.9211	0.046	4.583	89
M8	3514.0265	0.923	0.045	4.635	89, 195
M8a	3519.5486	0.7351			
M1a v. M2a: df = 2, -2Δln*L *= 11.046, **p < 0.001**					
M8 v. M8a: df = 1, -2Δln*L *= 11.044, **p < 0.001**					
					
*All delphinids:*					
M1a	2251.6189	0.548			
M2a	2236.8709	0.9868	0.028	14.257	20, 89, 195
M7	2251.7481	0.5			
M8	2236.871	0.9887	0.045	14.308	20, 89, 195
M8a	2251.6189	0.5479			
M1a v. M2a: df = 2, -2Δln*L *= 29.496, **p < 0.001**					
M8 v. M8a: df = 1, -2Δln*L *= 29.496, **p < 0.001**					
					
*All mysticetes:*					
M1a	1690.448	1			
M2a	1688.2783	1.5764	0.014	41.761	None
M8	1690.448	1.5764	0.014	41.757	None
M8a	1690.448	1			
M1a v. M2a: df = 2, -2Δln*L *= 4.339, p = 0.114					
M8 v. M8a: df = 1, -2Δln*L *= 0, p = 1.0					
					
*All primates:*					
M1a	5103.5715	0.6245			
M2a	5098.7948	0.728	0.065	2.608	None
M8	5098.9315	0.7297	0.132	2.13	209, 309
M8a	5103.5982	0.6244			
M1a v. M2a: df = 2, -2Δln*L *= 9.553, **p = 0.008**					
M8 v. M8a: df = 1, -2Δln*L *= 9.333, **p = 0.005**					
					
*No cetaceans/primates:*					
M1a	8990.4108	0.7309			
M2a	8987.9053	0.8132	0.059	2.17	None
M8	8980.7366	0.7764	0.117	1.82589	None
M8a	8986.2851	0.7017			
M1a v. M2a: df = 2, -2Δln*L *= 5.011, p = 0.082					
M8 v. M8a: df = 1, -2Δln*L *= 11.010, **p = 0.002**					

Each data set is listed separately with models, likelihood score (-ln*L*), average ω, the proportion of sites in the site class with ω > 1, the ω estimate for the site class with ω > 1, and the specific sites with ω > 1 using the Bayes empirical Bayes (BEB) procedure. Likelihood ratio tests for site models are also shown below for each data set with degrees of freedom (df), likelihood ratio (-2Δln*L*), and *p*-value. Statistically significant *p*-values are shown in bold.

We performed further tests to determine whether evidence for positive selection was restricted to different subclades of our tree, or alternatively whether positive selection is a general feature that characterizes the evolution of *MCPH1 *in the mammalian lineages sampled here. According to models M2a and M8, clades that showed strong evidence for positive selection acting at a subset of sites included Primates, Cetacea, Odontoceti (species tree only), Delphinidae, and all mammals excepting cetaceans and primates (M8 only) (Table 1). Both site models did not indicate positive selection within Mysticeti, but only four mysticete sequences were sampled here, and this is well below the recommended number for robust tests of positive selection using models M2a and M8 [38].

For the exon 8 data set, several branch model comparisons were executed (Table 2). We compared model M0 (one ω across the whole tree) to the free-ratio model in which all branches had separate ω values; the parameter rich free-ratio model was not a significantly better fit than M0 (*p *= 0.464; Table 2). Of the two-branch models explored here (see Methods), three "foreground" branches or sets of foreground branches showed evidence of ω greater than 1: the branch leading to the LCA of Mysticeti (ω = 1.340; ω = 3.115 for the gene tree), the set of all mysticete branches (ω = 1.838), and the terminal branch that connects to *Physeter *(ω = 1.447). Although ω was high for foreground branches in these various two branch models, fit was not significantly better than model M0 (one ω) following Bonferroni corrections. In mysticetes, ω was generally high but not significantly greater than 1 according to the LRT (Table 2).

**Table 2:** Branch models

Model	-ln *L*	*p *(GT)	ω (GT)	-ln *L*	*p *(ST)	ω (ST)	
Cetacea	15867.095	0.534	0.963	15996.857	0.545	0.962	
Mysticeti	15865.373	0.050	3.115	15996.264	0.213	1.340	
Odontoceti	15867.250	0.781	0.639	15997.024	0.858	0.767	
*Physeter*	15867.035	0.477	1.328	15996.698	0.408	1.447	
Delphinoidea	15867.288	0.994	0.768	15997.023	0.854	0.785	
Delphinidae	15867.268	0.840	0.730	15996.491	0.295	0.440	
*Orcinus*	15866.947	0.409	0.428	15996.963	0.695	0.613	
All cetaceans	15865.290	0.046	0.940	15995.134	0.051	0.930	
All mysticetes	15864.062	0.011	1.840	15993.772	0.011	1.838	
All odontocetes	15866.791	0.319	0.861	15996.583	0.340	0.854	
All delphinoids	15867.261	0.816	0.789	15997.020	0.840	0.759	
All delphinids	15867.026	0.469	0.899	15996.952	0.675	0.829	
Free ratio	15797.540	0.310		15929.589	0.464		

These are listed with likelihood score (-ln *L*), *p*-value of likelihood ratio test v. M0, and ω for the foreground branches when applicable. Results are shown for analyses using the *MCPH1 *gene tree as well as the species tree.

### *MCPH1 *Polymorphism in Cetacea

A total of 38 sites in exon 8 were heterozygous in at least one of the cetacean species sampled here; 14 species showed allelic variation. Parsimony optimization of this variation onto the *MCPH1 *gene tree (Figure 2) suggests that 22 nonsynonymous and 18 synonymous changes can account for the intraspecific variation (28 transitions and 12 transversions). None of the 40 point mutations included changes to a stop codon. Ten changes were at first codon positions, 10 at second positions, and 20 at third positions; some of the allelic differences were shared among closely related species in our sample (e.g., between *Delphinus delphis *and *D. capensis*).

A comparison between substitutions that characterize change among cetacean species and polymorphic mutations within cetacean species indicated that a greater proportion of substitutions among species were nonsynonymous than within species (326 nonsynonymous and 154 synonymous estimated using model M0 for the species tree of [42] versus 22 nonsynonymous and 18 synonymous changes within species). The larger proportion of nonsynonymous change across the cetacean phylogeny (68%) relative to the proportion of nonsynonymous mutations within species (55%) is consistent with positive selection [48], but much more extensive sampling of *MCPH1 *variation within species is necessary to rigorously test this hypothesis in the future.

### Association of ω with Phenotype

We explicitly tested the hypothesis that selection intensity on *MCPH1*, as represented by root-to-tip ω (Additional File 1: Table S2), was positively correlated with different measures of brain and body size in cetaceans. Based on phylogenetically controlled regression analyses, we found no significant association between ω and absolute brain mass or EQ (Table 3). There is a significant association with absolute body mass, (p = 0.024; Table 3) and a non-significant trend with brain mass across all cetaceans, which suggests a closer relationship between ω and overall body size. However both regression coefficients are low, and when mysticetes are excluded the associations become weaker, implying that these large-bodied species have a major effect on results (Table 3). To further explore the relationship between selection on *MCPH1 *and phenotypic evolution, we performed a multiple regression with root-to-tip *dN *and root-to-tip *dS *(log-transformed) for both absolute body and brain mass across cetaceans, in order to partition out the effects of *dN *and *dS. dN *was not significantly positively associated with either phenotype (body mass: t_23 _= 0.803, p = 0.215; brain mass t_23 _= 0.521, p = 0.304), whereas *dS *was significantly negatively associated with both (body mass: t_23 _= 2.498, p = 0.010; brain mass t_23 _= 2.022, p = 0.027). The negative relationship between *dS *and body mass is consistent with the conclusions of previous studies which show that the neutral rate of molecular evolution is associated with body mass and life history traits such as generation time and life span [49,50]. Hence, we cannot rule out the possibility that the weak associations observed reflect variation in the neutral substitution rate, which is associated with body size, rather than adaptive evolution.

**Table 3:** Regression analyses

A)						
Analysis	n	R^2^	t-statistic	p-value (1 tailed positive)		
*All Cetaceans*						
ω vs. brain mass	27	0.100	1.663	0.054		
ω vs. EQ	27	0.105	-1.710	1.000		
ω vs. body mass	27	0.148	2.087	0.024		
ω vs. max body mass	27	0.112	1.776	0.044		
						
*Toothed Whales only*						
ω vs. brain mass	25	0.064	1.249	0.112		
ω vs. EQ	25	0.031	-0.860	1.000		
ω vs. body mass	25	0.079	1.408	0.086		
ω vs. max body mass	25	0.084	1.454	0.079		

**B)**			**dN**		**dS**	
			
**Analysis**	**n**	**R^2^**	**t-statistic**	**p-value** **(1 tailed positive)**	**t-statistic**	**p-value** **(1 tailed negative)**

*All Cetaceans*						
Body mass vs. *dN *&*dS*	27	0.231	0.803	0.215	-2.498	0.010
Brain mass vs. *dN *&*dS*	27	0.173	0.521	0.304	-2.022	0.027

A) Regression analyses using BayesTraits of root-to-tip ω (*dN/dS*) versus absolute brain mass, relative brain mass (encephalization quotient = EQ), absolute body mass, and maximum body mass derived from [45]. Results are shown for all cetaceans and toothed whales only. B) Multiple regression analyses of absolute brain mass and absolute body mass versus *dN *(rate of nonsynonymous substitution) and *dS *(rate of synonymous substitution).

## Discussion

### *MCPH1 *as a Phylogenetic Marker in Mammals

Despite, or because of its molecular evolutionary dynamics, exon 8 of *MCPH1 *performed well as a phylogenetic marker at both deep and shallow nodes (Figure 2; Additional File 1: Figure S1, Figure S2). Most relationships among mammalian orders and supraordinal clades in the *MCPH1 *tree are congruent with analyses supported by much larger data sets [39,40], and 15 clades within Primates are characterized by high support scores and congruence with comprehensive phylogenetic hypotheses for this group [41,51] (Additional File 1: Figure S2). We also obtained good resolution within Cetacea (Figure 2), as compared to a recent supermatrix analysis of data from over 50 genes [42]. Overall, the congruence of our *MCPH1 *topology (Figure 2; Additional File 1: Figure S1, Figure S2) with published results is impressive, and is consistent with some previous studies which showed that highly variable nuclear genes with extensive amino acid replacements and evidence of positive selection can be efficient phylogenetic markers [52,53].

### The Evolution of *MCPH1 *across Mammals

Cetaceans, especially odontocetes, display multiple neuroanatomical and behavioral similarities with primates. Representatives of both groups have evolved large brains relative to their body sizes as well as highly complex cognitive abilities [9,10], although some researchers dispute this latter point [13]. In addition, cetaceans show similarities with great apes in brain histology, neural connectivity, and enlargement of specific parts of the brain associated with cognition and social awareness [54], as well as extensive gyrification, or folding of the cerebral cortex [10,54]. Due to their convergence in multiple neurological and behavioral features, cetaceans present an obvious test of the hypothesis that *MCPH1 *is related to the evolution of large brain size in mammals.

Analysis of the full coding sequence of *MCPH1 *(Figure 3) revealed high ωs on the stem branch to hominid primates and also on the terminal branch leading to the bottlenose dolphin *Tursiops*. Very high average ω over the entire protein-coding sequence of a gene, especially one this long (~2500 bp), is rare, as indicated by comparisons across complete genome sequences of different species (e.g., [55]). The high ω on the cetacean branch, in combination with the superior fit of positive selection models to the *MCPH1 *data relative to nearly neutral models, suggests a persistent signal of positive selection since the bottlenose dolphin lineage split from *Bos taurus *(Ruminantia). Comparative analyses of *MCPH1 *sequences did not reveal evidence for precise site-specific convergent molecular evolution of *MCPH1 *between cetaceans and primates at the amino acid level in either the whole gene or exon 8 data sets. However for exon 8, seven sites were identified as evolving under positive selection in Boreoeutheria (Table 1). Changes at positively selected sites, inferred using the site models, were not restricted to either Primates or Cetacea suggesting that the gene may have an important evolutionary role across mammals (Table 1). Further detailed analysis of other mammalian clades will be needed to determine how extensive positive selection has been on *MCPH1*.

### The Evolution of *MCPH1 *within Cetacea and its Relation to Brain Size

With the inclusion of more species in the exon 8 data set, estimated ω ratios revealed a more complex pattern of evolution in cetaceans. In general, exon 8 had a relatively high mean ω across mammals (Table 1) and ω within Cetacea was elevated relative to the remaining branches in the tree (Table 2). Both Odontoceti and Delphinoidea represent clades in which shifts to larger relative brain sizes have been proposed, and the highest EQs are restricted to members of Delphinidae [10]. However, our comparative analyses of *MCPH1 *did not reveal especially high ω scores on the branches leading to the LCAs of Cetacea, Odontoceti, Delphinoidea, or Delphinidae (Table 2). Absence of strong evidence for positive selection in these lineages does not match the evolutionary change of the EQ in which there is a large increase in relative brain size along the lineage leading from the LCA of Cetacea to the LCA of Delphinidae [10]. Regression analyses agreed with these results, finding no robust associations between ω and absolute or relative brain size (Table 3).

Some of the largest ω scores, according to the branch models, were recorded along the branch leading to the LCA of mysticetes and across all mysticete branches (Table 2). This is contrary to the hypothesis that selection in *MCPH1 *is related to an increase in relative brain size [16,19], as mysticetes have smaller brains than expected given their body size compared to the mammalian average [2]; however, it does not rule out that *MCPH1 *may be linked to overall change in EQ within cetaceans. Although mysticetes have low EQs, the brains of these cetaceans have the largest absolute mass in the animal kingdom, aside from elephants and the odontocete *Physeter macrocephalus *(giant sperm whale; Figure 1) [10]. It has been argued that the molecular evolution of genes involved in the proliferation of neural progenitor cells should bear a closer relation to absolute brain size than relative brain size [25]; if *MCPH1 *has had such an evolutionary role we may then expect it to have high rates of evolution during the evolution of the largest cetaceans (Figure 1). Both the ancestral mysticete branch and the *Physeter *terminal branch have high ω (Table 2), but the branch leading to the very large delphinid, *Orcinus orca *which dwarfs most extant and extinct oceanic dolphins (Figure 1), did not show an extreme ω for *MCPH1 *in comparison to dolphins with much smaller brain and body sizes (Table 2; Additional File 1: Table S2). Our regression analyses do not support a robust association of ω with absolute brain mass, so we cannot confidently say that the high ω in mysticetes is causally related to selection for large brain size. Indeed, a stronger association was found between root-to-tip ω and body mass and therefore we cannot exclude body mass, or other correlated traits, from being the relevant phenotype. Our multiple regressions with *dN *and *dS *furthermore suggest that these trends may be driven largely by variation in *dS*, instead of *dN*, perhaps related to variation in life history traits which affect the rate at which neutral variants are fixed [49,50].

The lack of association between brain size and the evolution of *MCPH1 *found in cetaceans agrees with the conclusions drawn from a recent study of anthropoid primates which showed that *MCPH1 *is not associated with the evolution of either absolute or relative brain size [25], despite the clear critical importance of *MCPH1 *in brain development. Together these studies question the commonly held assumption that this locus has a direct role in the evolution of brain size as a gross measure [16,19,56]. While we cannot rule out more nuanced roles in brain evolution, the phenotypic relevance of positive selection on this locus is currently an outstanding issue.

*MCPH1 *is expressed in many other tissues [32], and mutations in *MCPH1 *have been discovered in cancerous tumors [27]. Some mysticetes have very long lifespans [57], are the largest animals that have yet evolved, and are characterized by a very high fetal growth rate [58]. Given that *MCPH1 *functions in cell cycle regulation and DNA damage repair [24], it is plausible that positive selection in mysticetes and other mammals is related to the evolution of other phenotypes such as the rate of growth, absolute number of cells, or tumor suppression in very large organisms with long lifespans, although these hypotheses are currently speculative. Some evidence, presented here, suggests that positive selection has acted on this locus in non-primate, non-cetacean mammals (Table 1 - model M8). It may therefore be possible to study the evolutionary function of MCPH1, and to test these hypotheses, in more experimentally tractable organisms such as rodents.

## Conclusions

Extant cetacean species are characterized by a very broad range of body sizes and brain sizes, among the greatest variation seen in any mammalian order (Figure 1). Although this study did not find evidence for a statistically significant association between selection intensity (ω) in *MCPH1*, a gene associated with microcephaly, and absolute or relative brain size in cetaceans (Table 3), some intriguing patterns emerged from detailed phylogenetic and molecular analyses of *MCPH1*. Evolutionary models that included parameters for positive selection consistently fit the *MCPH1 *sequence data significantly better than alternative models of negative selection and neutral change (Table 1). Using complete sequences of *MCPH1*, we identified ω > 1 in only two cases, along the lineage leading to *Tursiops *(bottlenose dolphin) and also on the branch leading to Hominidae (human, gorilla, chimp, and orang). Furthermore, analysis of a larger data set of cetacean sequences derived from the hypervariable exon 8 again indicated a very high average ω within Cetacea, with the largest ω values in Mysticeti, a group characterized by low EQ but large absolute brain and body weights. Despite finding strong evidence for positive selection having acted on a gene which has a key role in brain development, we found no compelling evidence to support the hypothesis that there is an association between the evolution of this locus and the evolution of brain size in cetaceans. This is in agreement with results from anthropoid primates, and suggests that positive selection on *MCPH1 *may be related to change in phenotypes other than gross brain size.

## Authors' contributions

MM, CC, and JG gathered *MCPH1 *sequence. MM aligned sequences and conducted phylogenetic analyses. MM and SM executed PAML analyses. SM conducted regression analyses. MM wrote the paper, and all authors read, edited, and approved the final manuscript.

## Supplementary Material

Additional file 1 **Supplemental Information**. This includes Supplemental Methods, Supplemental Results, Supplemental Appendix I, Supplemental Tables S1, S2, S3 and Supplemental Figures S1, S2. Click here for file
