# Supplementary material for: Phylogeny and adaptive evolution of the brain-development gene microcephalin (MCPH1) in cetaceans
Source: BMC Evol Biol. 2011 Apr 14;11:98. doi: 10.1186/1471-2148-11-98 (PMC3101173; doi:10.1186/1471-2148-11-98)
Supplement: Additional file 1 — Supplemental Information. This includes Supplemental Methods, Supplemental Results, Supplemental Appendix I, Supplemental Tables S1, S2, S3 and Supplemental Figures S1, S2. [file 1471-2148-11-98-S1.PDF]

ADDITIONAL FILE 1

SUPPLEMENTAL INFORMATION

Supplemental Methods

Phylogenetic Analysis

Phylogenetic analyses were performed on the exon 8 data set only. Maximum-likelihood (ML) searches were conducted in RAxML 7.2.0 [59] via the Cyberinfrastructure for Phylogenetic Research (CIPRES) Portal (www.phylo.org). Insertion/deletion (indel) characters were excluded, as RAxML does not implement binary models of character evolution in conjunction with nucleotide substitution models. We conducted analyses using four data partitions, one partition for the intron and three partitions that account for the three codon positions. The Akaike Information Criterion (AIC) was used to choose the optimal partitioning strategy. We used standard default parameters, including the GTRMIX option with the GTR+G model of nucleotide evolution. To assess support, one-thousand ML bootstrap replicates also were calculated in RAxML using the CIPRES interface [60].

Markov chain Monte Carlo (MCMC) Bayesian analyses were executed using default parameters and four simultaneous chains (three “heated,” one “cold”) in MrBayes 3.1.2 [61] via CIPRES. We used the same partitioning strategies as with RAxML, and conducted analyses with and without indel characters. The simple gap coding procedure of [62] was utilized as implemented in the program SeqState [63], and indels were treated as a separate partition using the binary model of character evolution [64]. For DNA sequence alignments, MrModeltest 2.2 [65] was employed to choose optimal models for

each data partition according to the AIC [66] (intron: GTR, all other partitions: HKY+G). Two concurrent runs of 20,000,000 generations were employed for each analysis with trees sampled every 1000 generations to assess convergence. The stationarity of likelihood scores for sampled trees was assessed in Tracer v1.04 [67], and the standard deviation of split frequencies between runs was evaluated using AWTY [68] to establish that concurrent runs had converged. The first 20% of trees were discarded as burn-in, and a 50% majority rule consensus of the remaining trees was constructed from both runs to summarize posterior probabilities for each clade.

Supplemental Results

Characterization of *MCPH1*

The total alignment of the "exon 8 data set" was 1377 bp with 1308 bp in exon 8 and the first 69 bp assigned to the preceding intron 7. Total number of indels was 54, ranging from 1-102 bp in length. Protein translations of the sequenced portion of exon 8 ranged from 364 aa in *Rattus* to 415 aa in *Felis*, and within cetaceans, 381 aa to 384 aa.

In the exon 8 data set, both sequences from Monodontidae (*Monodon* and *Delphinapterus*) possessed a nucleotide substitution C1371T that resulted in a stop codon near the 3' end of exon 8. All other changes in exon 8 of cetaceans, 364 substitutions and six indels according to parsimony reconstructions, did not result in stops or frameshifts. The single stop codon in monodontids would produce a truncated MCPH1 protein that lacks two BRCA1 C-terminal (BRCT) domains that are critical in regulation of DNA repair prior to mitosis [69]. Mutations causing a loss of the C-terminal BRCT domains yield a microcephaly phenotype in humans [70], but do not seem to affect brain size in

mice, although other abnormalities are present [71]. Both *Monodon* and *Delphinapterus* have encephalization quotients (1.76 and 2.24, respectively) [8] that are within the range of other odontocete whales, and if *MCPH1* were associated with brain size it is unlikely that truncation occurred in these species. There is no evidence that the monodontid sequences represent paralogous pseudogenes, as there were no obvious deviations in rate of evolution or expected phylogenetic placement of the sequences (see below). The proximity of the stop codon to an exon-intron boundary, and another substitution (A1341G) upstream from the stop codon in Monodontidae suggests the possibility of a change in exon-intron boundary. However, this hypothesis is speculative. As stated above, for all species in the PAML runs of the exon 8 data set, we excluded the three bases that are homologous to the monodontid stop codon and all downstream nucleotides. In addition, we also performed PAML analyses using the site models with the monodontid sequences excluded (reduced exon 8 data set).

Phylogenetic Results

Most higher-level relationships among mammalian orders and suborders were consistent with those of large comprehensive data sets including Laurasiatheria, Euarchontoglires, Rodentia, Scandentia + Primates, Primates, Carnivora, Cetartiodactyla (=Artiodactyla), Cetruminantia, and Cetacea [39, 40]. Relationships within Primates were generally congruent with the most recent and inclusive phylogenetic treatment of the group including Strepsirrhini, Anthropoidea, Catarrhini, Platyrrhini, Hominoidea, Hominidae, Cercopithecidae, Hylobatidae, Cercopithecinae, Colobinae, Homininae, Hominini, and *Macaca* [41]. Relationships within Cetacea supported by *MCPH1* (Fig. 2)

included Mysticeti, paraphyly of Balaenopteridae with *Balaenoptera acutorostrata* basal, Odontoceti, Physeteroidea, Ziphiidae + Delphinoidea, Ziphiidae, *Mesoplodon* +*Ziphius* (to the exclusion of *Berardius)*, Delphinoidea, Monodontidae + Phocoenidae, Monodontidae, Phocoenidae, Delphinidae, Lissodelphininae, monophyly of *Sagmatias*, Globicephalinae, *Globicephala* + *Peponocephala, Globicephala* + *Peponocephala* + *Feresa, Globicephala* + *Peponocephala* + *Feresa* + *Pseudorca, and Sotalia* + Delphininae; all of these results were also supported by the supermatrix of [42]. Some relationships among cetaceans differed from recent multigene analyses [42, 72, 73], including the grouping of *Platanista* + Physeteroidea, *Eschrichtius* + *Megaptera*, and *Neophocaena* + *Phocoenoides*, although support scores for these nodes were very low (Supplemental Fig. 1). The *MCPH1* topology positioned *Leucopleurus acutus* as the sister-taxon to all other delphinids in the analysis; this differs from [42, 73], which both placed *Orcinus orca* as more basal than this taxon. The *MCPH1* gene tree grouped *Orcinus* with *Lagenorhynchus albirostris* and *Steno bredanensis* in a poorly-supported, novel clade. Low support for many subclades of Delphinidae reflected the difficulty of resolving the branching sequence in this group, as evidenced by recent analyses of multigene concatenations [42, 73, 74] and amplified fragment length polymorphisms [75]. Both species with sequences from two individuals (*Delphinus capensis* and *Tursiops truncatus*) were not recovered as monophyletic, but these results again were very weakly supported (Fig. 2; Supplemental Fig. 1).

LITERATURE CITED

59 Stamatakis A: **RAxML-VI-HPC: Maximum likelihood-based phylogenetic analyses with thousands of taxa and mixed models.** *Bioinformatics* 2006, **22**: 2688-2690.
60 Stamatakis A, Hoover P, Rougemont J: **A rapid bootstrap algorithm for the RAxML web servers**. *Syst Biol* 2008, **57**:758-771.
61 Ronquist F, Huelsenbeck JP: **MRBAYES 3: Bayesian phylogenetic inference under mixed models.** *Bioinformatics* 2003, **19**:1572-1574.
62 Simmons MP, Ochoterena H: **Gaps as characters in sequence-based phylogenetic analyses.** *Syst Biol* 2000, **49**:369-381.
63 Müller K: **SeqState - primer design and sequence statistics for phylogenetic DNA data sets.** *Appl Bioinformat* 2005, **4**:65-69.
64 Ronquist F, Huelsenbeck JP, van der Mark P: **MrBayes 3.1 Manual Draft 5/26/2005**, 2005 [http://mrbayes.csit.fsu.edu/manual.php].
65 Nylander JAA: **MrModeltest v2**. Program distributed by the author. Evolutionary Biology Centre, Uppsala University; 2004.
66 Posada D, Buckley TR: **Model selection and model averaging in phylogenetics: advantages of Akaike Information Criterion and Bayesian approaches over likelihood ratio tests.** *Syst Biol* 2004, **53**:793-808.
67 Rambaut A, Drummond AJ: **Tracer v1.4**, 2004 [http://beast.bio.ed.ac.uk/Trace].
68 Nylander JAA, Wilgenbusch JC, Warren DL, Swofford DL: **AWTY (are we there yet?): a system for graphical exploration of MCMC convergence in Bayesian phylogenetics.** *Bioinformatics* 2008, **24**:581-583.
69 Jeffers LJ, Coull BJ, Stack SJ, Morrison CG: **Distinct BRCT domains in Mcph1/Brit1 mediate ionizing radiation-induced focus formation and centrosomal localization.** *Oncogene* 2008, **27**:139-144.
70 Trimborn M, Bell SM, Felix C, Rashid Y, Jafri H, Griffiths PD, Neumann LM, Krebs A, Reis A, Sperling K, Neitzel H, Jackson AP: **Mutations in *Microcephalin* cause aberrant regulation of chromosome condensation.** *Am. J. Hum. Genet.* 2004, **75**:261-266.
71 Trimborn M, Ghani M, Walther DJ, Dopatka M, Dutrannoy V, Busche A, Meyer F, Nowak S, Nowak J, Zabel C, Klose J, Esquinito V, Garshasbi M, Kuss AW, Ropers HH, Mueller S, Poehlmann C, Gavvovidis I, Schindler D, Sperling K, Neitzel H: **Establishment of a mouse model with misregulated chromosome condensation due to defective *Mcph1*.** *PLoS ONE* 2010, **5**:e9242.
72 Deméré TA, McGowen MR, Berta A, Gatesy J: **Morphological and molecular evidence for a stepwise evolutionary transition from teeth to baleen in mysticete whales.** *Syst Biol* 2008, **57**:15–37.
73 Steeman ME, Hebsgaard MB, Fordyce RE, Ho SYW, Rabosky DL, Nielsen R, Rahbek C, Glenner H, Sørenson MV, Willerslev E: **Radiation of extant cetaceans driven by restructuring of the oceans.** *Syst Biol* 2009, **58**:573-585.
74 Caballero S, Jackson J, Mignucci-Giannoni AA, Barrios-Garrido H, Beltrán-Pedreros S, Montiel-Villalobos MG, Robertson KM, Baker CS: **Molecular systematics of South American dolphins *Sotalia*: sister taxa determination and phylogenetic relationships, with insights into a multi-locus phylogeny of the Delphinidae.** *Mol Phylogenet Evol* 2008, **46**:252-268.

75 Kingston SE, Adams LD, Rosel PE: **Testing mitochondrial sequences and anonymous nuclear markers for phylogeny reconstruction in a rapidly radiating group: molecular systematics of the Delphininae (Cetacea: Odontoceti: Delphinidae).** *BMC Evol Biol* 2009, **9**:245.

SUPPLEMENTAL APPENDIX I.

List of non-cetacean species and respective Genbank accession numbers (if applicable) of *MCPH1* sequences used in this study: *Homo sapiens* (Ensembl), *Pan troglodytes* (Ensembl), *Gorilla gorilla* (Ensembl), *Pongo pygmaeus* (AY307815), *Hylobates lar* (AY553031), *Nomascus leucogenys* (AY307841), *Hoolock hoolock* (AY505993), *Macaca mulatta* (Ensembl), *Macaca fascicularis* (AY742816), *Rhinopithecus bieti* (AY307843), *Pygathrix nemaeus* (AY505995), *Colobus guereza* (AY553045), *Trachypithecus francoisi* (AY505996), *Erythrocebus patas* (AY505994), *Callithrix jacchus* (Ensembl), *Lagothrix lagotricha* (AY533018), *Saimiri boliviensis* (AY570949), *Otolemur garnettii* (Ensembl), *Lemur catta* (AY570945), *Tupaia belangeri* (Ensembl), *Mus musculus* (Ensembl), *Rattus norvegicus* (Ensembl), *Equus caballus* (Ensembl), *Canis familiaris* (Ensembl), *Felis catus* (Ensembl), *Sus scrofa* (Ensembl), *Bos taurus* (Ensembl), *Loxodonta africanus* (Ensembl).

Table S1. List of taxa sampled with specimen IDs. (All specimen ID numbers beginning with "Z" originated from Southwest Fisheries Science Center; HR=Howard Rosenbaum; MM=Michel Milinkovitch; MS=Michael Stanhope; PP=Per Palsbøll).

Taxa	Specimen ID
Mysticeti	
Balaenopteridae	
Balaenoptera acutorostrata (minke whale)	Z13091
Balaenoptera physalus (fin whale)	HR
Megaptera novaeangliae (humpback whale)	Z11727
Eschrichtiidae	
Eschrichtius robustus (gray whale)	Z13090
Odontoceti	
Physeteridae	
Physeter macrocephalus (giant sperm whale)	MM
Kogiidae	
Kogia sima (dwarf sperm whale)	Z10124
Platanistidae	
Platanista minor (Indian river dolphin)	Z15224
Ziphiidae	
Berardius bairdii (Baird's beaked whale)	Z4963
Mesoplodon bidens (Sowerby's beaked whale)	Z3859
Ziphius cavirostris (Cuvier's beaked whale)	Z2157
Monodontidae	
Delphinapterus leucas (beluga whale)	Z35275
Monodon monoceros (narwhal)	PP
Phocoenidae	
Neophocaena phocaenoides (finless porpoise)	Z984
Phocoena phocoena (harbor porpoise)	Z28452
Phocoenoides dalli (Dall's porpoise)	Z38979
Delphinidae	
Cephalorhynchus commersonii (Commerson's dolphin)	Z480
Delphinus capensis (long-beaked common dolphin)	Z23185
Delphinus capensis (long-beaked common dolphin)	Z26654
Delphinus delphis (short-beaked common dolphin)	Z31912
Feresa attenuata (pygmy killer whale)	Z3944
Globicephala macrorhynchus (short-finned pilot whale)	Z39091
Grampus griseus (Risso's dolphin)	Z483
Lagenorhynchus albirostris (white-beaked dolphin)	Z17318
Lagenodelphis hosei (Fraser's dolphin)	Z30468
Leucopleurus acutus (Atlantic white-sided dolphin)	Z7842
Lissodelphus borealis (Northern right-whale dolphin)	Z39099
Orcinus orca (killer whale)	Z6004
Peponocephala electra (melon-headed whale)	Z34003
Pseudorca crassidens (false killer whale)	Z38069
Sagmatias obliquidens (Pacific white-sided dolphin)	Z25409
Sagmatias obscurus (dusky dolphin)	Z37807
Sotalia fluviatilis (tucuxi)	MS-T1826
Sousa chinensis (Indo-Pacific humpback dolphin)	HR-OM41
Stenella attenuata (pantropical spotted dolphin)	Z18473
Stenella coeruleoalba (striped dolphin)	Z1571
Stenella longirostris (spinner dolphin)	Z24045
Steno bredanensis (rough-toothed dolphin)	Z38282
Tursiops aduncus (Indo-Pacific bottlenose dolphin)	Z4172
Tursiops truncatus (common bottlenose dolphin)	Z38274

Table S2. Comparative data used in regression analyses.

Species	EQ	Brain mass (g)	Body mass (g)	Max body mass (g)	Log(EQ)	Log (Brain mass)	ω	log(ω)	Source
Delphinus delphis	4.27	814.3	59980.7	200000	0.630335464	2.910784435	0.6531	-0.185020316	[8]
Tursiops truncatus	4.02	1759.2	206823.8	650000	0.604681626	3.245315216	0.6846	-0.164563105	[8]
Stenella longirostris	3.22	660	66200	82000	0.50776246	2.819543936	0.6243	-0.204606665	[8]
Stenella coeruleoalba	2.95	938.5	128715.6	156000	0.469800137	2.972434277	0.6639	-0.177897331	[8]
Sotalia fluviatilis	4.57	688	42200	40000	0.65944785	2.837588438	0.8002	-0.096801453	[8]
Steno bredanensis	4.97	1541.9	123830.9	155000	0.696679513	3.188056208	0.7117	-0.147703034	[8]
Lissodelphis borealis	5.34	1162	73000	115000	0.727598566	3.065206128	0.6304	-0.200383796	[44]
Sagmatias obscurus	4.72	886	58473.1	85000	0.674391876	2.947433722	0.6411	-0.193074223	[8]
Sagmatias obliquidens	5.27	1045	63500	198000	0.722076648	3.01911629	0.6411	-0.193074223	[8]
Leucopleurus acutus	2.25	1100.5	244217.1	235000	0.352598834	3.041590047	0.5794	-0.237021509	[8]
Feresa attenuata	3.71	1252	140000	225000	0.570517299	3.097604329	0.6636	-0.178093623	[44]
Cephalorhynchus commersonii	4.85	748	43666	86000	0.685824267	2.873901598	0.5735	-0.241466578	[44]
Globicephala macrorhynchus	3.71	2466	386000	3600000	0.569798332	3.391993072	0.7402	-0.130650919	[44]
Grampus griseus	4.07	2384.4	319974	500000	0.609771025	3.377379113	0.6755	-0.170374647	[8]
Pseudorca crassidens	4.03	3512	579196.4	2000000	0.605279939	3.545554507	0.6539	-0.184488663	[8]
Orcinus orca	2.56	5028	1953201	10000000	0.407413575	3.701395269	0.6241	-0.204745817	[8]
Phocoena phocoena	2.96	522.7	53510.9	70000	0.471014943	2.7182525	0.5876	-0.230918213	[8]
Phocoenoides dalli	3.55	861.4	85748.3	200000	0.550762524	2.935204867	0.5876	-0.230918213	[8]
Neophocaena phocaenoides	3.71	468	32400	72000	0.56899945	2.670245853	0.5934	-0.226652458	[43]
Delphinapterus leucas	2.24	2083	636000	1600000	0.351191756	3.31868927	0.8566	-0.06722193	[8]
Monodon monoceros	1.76	2993.7	1578116.9	1600000	0.244273787	3.476208277	0.7255	-0.139362583	[8]
Ziphius cavirostris	0.92	2004	2273000	3000000	-0.036205151	3.301897717	1.3082	0.116674145	[8]
Platanista sp.	1.50	295	63000	85000	0.175082602	2.469822016	0.9596	-0.017909761	[43]
Physeter macrocephalus	0.58	7999.4	35632153.8	57000000	-0.235858022	3.903057414	1.1253	0.051268319	[8]
Kogia sima	1.63	621.5	167958.3	272000	0.213374902	2.793441133	0.8968	-0.0473044	[8]
Megaptera novaeangliae	0.44	6439	39311330	40000000	-0.358689705	3.808818425	1.8087	0.257366539	[9]
Balaenoptera physalus	0.54	7085	33221430	120000000	-0.268191668	3.850339855	1.5393	0.187323269	[9]

Table S3. Results for site model analyses using the *MCPH1* gene tree. Each data set is listed separately with models, likelihood score (-ln*L*), average ω, the proportion of sites in the site class with ω>1, the ω estimate for the site class with ω>1, and the specific sites with ω>1 using the Bayes empirical Bayes (BEB) procedure. Likelihood ratio tests for site models are also shown below for each data set with degrees of freedom (df), likelihood ratio (-2Δln*L*), and *p*-value. Statistically significant *p*-values are shown in bold.

**Model**	**-ln *L***	**Avg. ω**	**Prop. sites ω>1**	**ω sites with ω>1**	**Sites under positive selection (p>0.95, BEB)**
All mammals:					
M1a	15755.134	0.758			
M2a	15744.237	0.855	0.07	2.074	362
M8	15739.018	0.828	0.14	1.709	112, 205, 209, 247, 353, 362
M8a	15751.423	0.738			
M1a v. M2a: df=2, -2Δln*L*=21.793, **p<0.001**					
M8 v. M8a: df=1, -2Δln*L*=24.810, **p<0.001**					
All cetaceans:					
M1a	3821.188	0.8519			
M2a	3817.018	0.9282	0.007	9.685	362
M8	3817.107	0.9722	0.03	4.777	362
M8a	3821.189	0.8529			
M1a v. M2a: df=2, -2Δln*L*=8.340, **p=0.015**					
M8 v. M8a: df=1, -2Δln*L*=8.164, **p=0.009**					
All odontocetes:					
M1a	3430.268	0.7866			
M2a	3428.656	0.9303	0.528	1.543	None
M8	3428.656	0.9304	0.528	1.544	None
M8a	3430.268	0.7866			
M1a v. M2a: df=2, -2Δln*L*=3.225, p=0.199					
M8 v. M8a: df=1, -2Δln*L*=3.224, p=0.100					
All delphinids:					
M1a	2174.555	0.7454			
M2a	2171.205	1.0042	0.052	7.393	None
M8	2171.205	1.0052	0.052	7.43	20, 89
M8a	2174.555	0.745			
M1a v. M2a: df=2, -2Δln*L*=6.700, **p=0.035**					
M8 v. M8a: df=1, -2Δln*L*=6.700, **p=0.018**					

All mysticetes:

M1a	1690.449	1			
M2a	1688.295	1.5764	0.013	43.568	None
M8	1688.295	1.5764	0.013	43.564	None
M8a	1690.449	1			

M1a v. M2a: df=2, -2Δln*L*=4.308, p=0.116

M8 v. M8a: df=1, -2Δln*L*=4.308, p=0.058

All primates:

M1a	5084.4735	0.6274			
M2a	5080.1506	0.7248	0.056	2.7174	None
M8	5080.3501	0.7258	0.121	2.1613	209, 309
M8a	5084.4913				

M1a v. M2a: df=2, -2Δln*L*=8.6458, **p=0.013**

M8 v. M8a: df=1, -2Δln*L*=8.2824, **p=0.002**

No cetaceans/primates:

M1a	8976.7747	0.7477			
M2a	8975.0353	0.8196	0.055	2.103	None
M8	8968.2834	0.7779	0.119	1.76	None
M8a	8972.7191	0.7115			

M1a v. M2a: df=2, -2Δln*L*=3.4788; p=0.1756

M8 v. M8a: df=1, -2Δln*L*=8.8714; **p=0.001**

Supplemental Figure Legends

Figure S1. Maximum-likelihood (ML) gene tree for *MCPH1* based on the exon 8 data set. Only cetaceans are shown. ML bootstrap scores are above each branch, below are Bayesian posterior probabilities of the Bayesian analysis with and without gaps (bottom).

Figure S2. Maximum likelihood gene tree for *MCPH1* based on the exon 8 data set. Cetacean branches are blue, and the remaining branches are black. Red dots on the phylogram mark nodes that have ML bootstrap ≥70% and Bayesian posterior probability (with and without indels) ≥ 0.95. High support scores within Cetacea are indicated in Figure 2.

Figure S1.

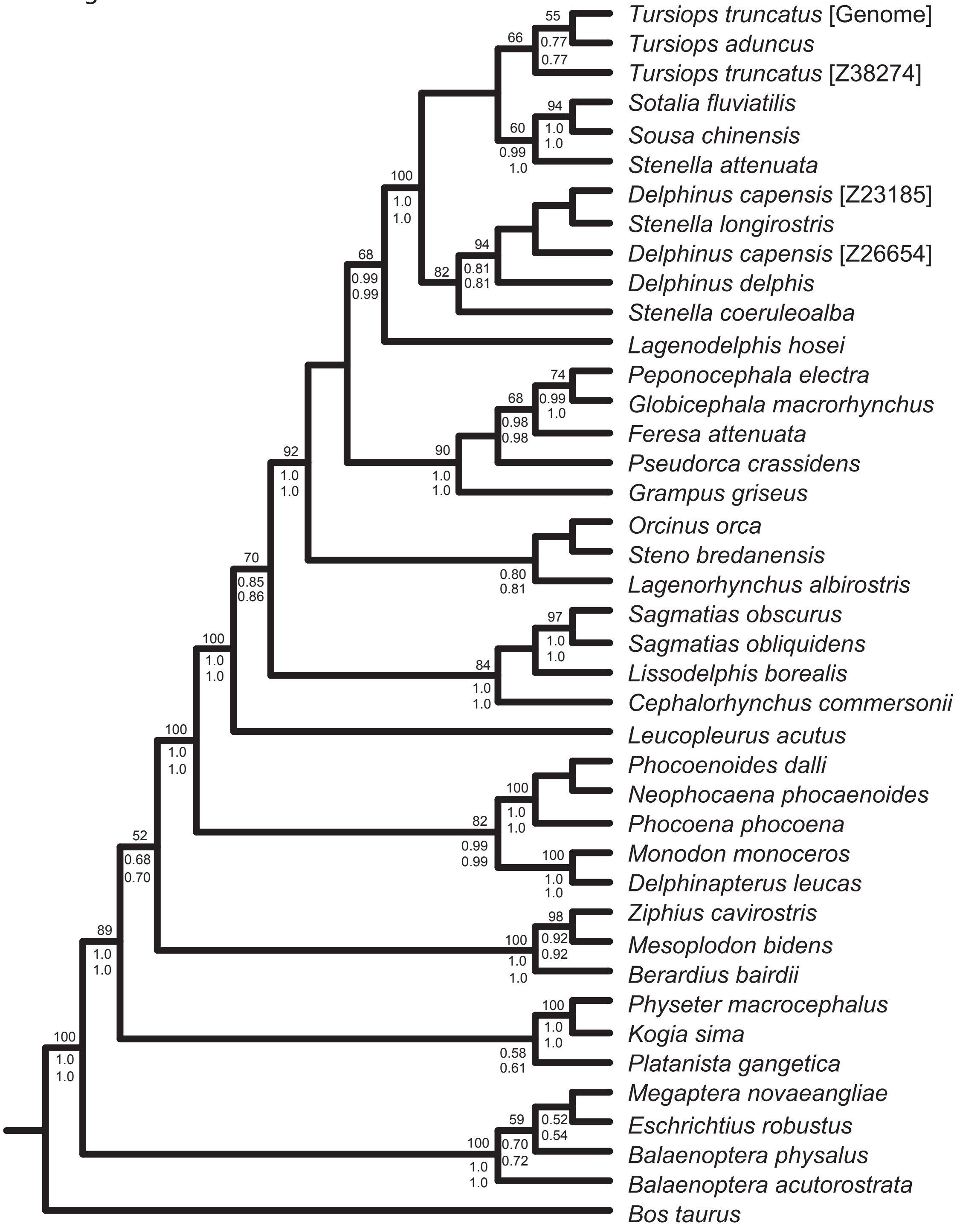
Tursiops truncatus [Genome]
Tursiops aduncus
Tursiops truncatus [Z38274]
Sotalia fluviatilis
Sousa chinensis
Stenella attenuata
Delphinus capensis [Z23185]
Stenella longirostris
Delphinus capensis [Z26654]
Delphinus delphis
Stenella coeruleoalba
Lagenodelphis hosei
Peponocephala electra
Globicephala macrorhynchus
Feresa attenuata
Pseudorca crassidens
Grampus griseus
Orcinus orca
Steno bredanensis
Lagenorhynchus albirostris
Sagmatias obscurus
Sagmatias obliquidens
Lissodelphis borealis
Cephalorhynchus commersonii
Leucopleurus acutus
Phocoenoides dalli
Neophocaena phocaenoides
Phocoena phocoena
Monodon monoceros
Delphinapterus leucas
Ziphius cavirostris
Mesoplodon bidens
Berardius bairdii
Physeter macrocephalus
Kogia sima
Platanista gangetica
Megaptera novaeangliae
Eschrichtius robustus
Balaenoptera physalus
Balaenoptera acutorostrata
Bos taurus

Figure S2.

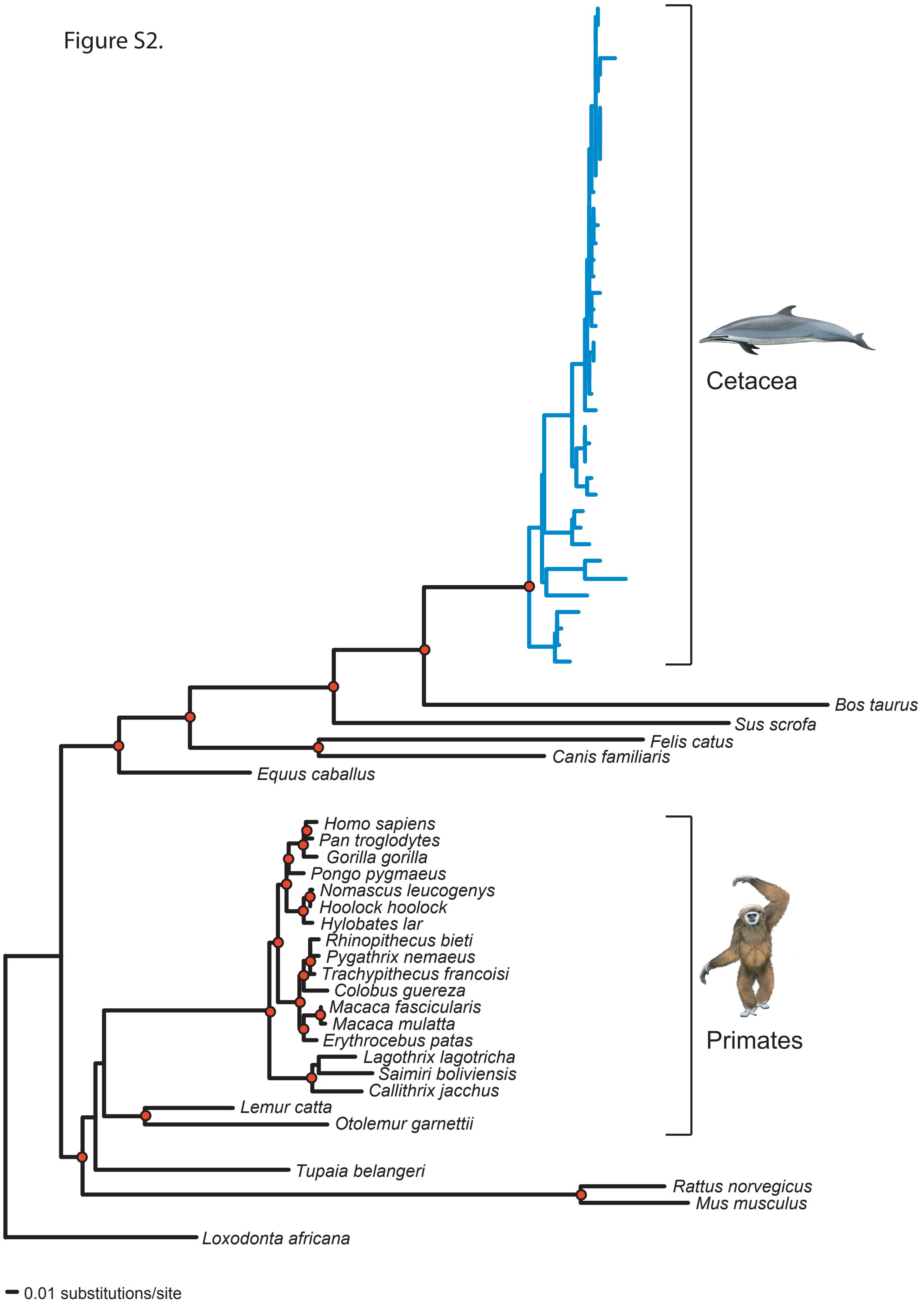
Cetacea
Bos taurus
Sus scrofa
Felis catus
Canis familiaris
Equus caballus
Homo sapiens
Pan troglodytes
Gorilla gorilla
Pongo pygmaeus
Nomascus leucogenys
Hoolock hoolock
Hylobates lar
Rhinopithecus bieti
Pygathrix nemaeus
Trachypithecus francoisi
Colobus guereza
Macaca fascicularis
Macaca mulatta
Erythrocebus patas
Lagothrix lagotricha
Saimiri boliviensis
Callithrix jacchus
Lemur catta
Otolemur garnettii
Primates
Tupaia belangeri
Rattus norvegicus
Mus musculus
Loxodonta africana
0.01 substitutions/site
